# The Importance of Examining the Teeth

**Published:** 1910-10-08

**Authors:** 


### Surgery.

### THE IMPORTANCE OF EXAMINING THE TEETH.

The practitioner is apt to regard the teeth as being the particular province of the dentist. This is a correct attitude, provided that his knowledge is sufficient to enable him to determine when the aid of the dentist should be sought. The latter can only treat cases when called upon to do so, and it frequently happens that irremediable mischief is done before he is consulted. The practitioner is in a position to give the necessary warning. He may, moreover, by judicious advice be the means of preventing disease, and thus obviating pain, inconvenience, and deformity.

### Development of the Jaws.

The development of the jaws is a complex question, but it may be assumed that two very important factors are the preservation of the temporary teeth and the determination of nutrition by the sufficient exercise of the masticatory muscles. Both these factors are regulated by the kind of food eaten by the child. People do not usually go to their dentist for instructions regarding their children's diet, and it therefore behoves the practitioner to be himself in a position to give sound advice on this question. Dr. Sim Wallace has constantly indicated the kind of diet that should be prescribed, and his attitude with regard to the relation that exists between dental caries and diet is summed up in the following quotation: "The cause of the present-day susceptibility to dental caries is that the natural foodstuffs are, to a large extent, deprived of their accompanying fibrous parts and prepared and consumed in a manner which renders them—especially the carbohydrates—liable to lodge and undergo acid fermentation in the mouth, while from the same cause and the induced conditions the acid-producing micro-organisms of the mouth lodge and multiply, and augment the rapidity and intensity of the acid fermentation." He pleads for the abolition of "pap" diet after the eruption of the temporary molars and for proper regulation of the foodstuffs, in order that work may be given to structures whose obvious mission is work, and that the teeth may be cleansed by natural means, as opposed to the artificial cleansing brought about by the toothbrush.

### The Milk Teeth.

It is by no means uncommon in private and hospital practice to hear the condition of temporary teeth regarded by parents and practitioners alike as a matter of no moment. That is an attitude that cannot be too strongly condemned. The association, so frequently found as to be almost constant, of enlarged and septic tonsils and adenoid growths in the noso-pharynx with curious teeth is so striking that one must regard the teeth as the probable primary focus. Certainly the condition of the mouth should receive attention before any operation on the throat is performed. Acute and chronic adenitis may be caused by carious teeth or neglected roots, and the tubercle bacillus may gain entrance by the same channels.

Parents frequently look upon the first permanent molar as a temporary tooth, and this mistake is easily understood when it is remembered that this tooth is the first permanent tooth to erupt, has no predecessor, and takes its place with the temporary teeth. It is a most important tooth, and should be carefully preserved.

### Pain and Toothache.

The frequency with which trigeminal neuralgia is found to originate from some dental lesion is well known. But the cause may not be obvious, and is often only revealed by careful investigation. Local pain may be entirely absent, thus making diagnosis more difficult. The patient, too, is on this account inclined to be sceptical as to the dental origin of the pain. The causative lesions are various, but a decomposing pulp in a tooth free from caries, a chronic pulpitis set up by an amalgam filling, a localised pyorrhœa alveolaris, and an apparently efficient crown are among the most obscure. A frequent site of referred pain is the ear, and many an earache has been cured by the extraction of septic roots. Trismus is a symptom not met with very frequently, but its presence should suggest the possible impaction of a lower wisdom tooth, for this is by far the commonest cause of this condition. There is, in connection with swellings about the mouth due to teeth, a popular superstition that nothing should be done till the swelling has gone down. Delay has been productive of much deformity, life has been endangered, and in a few cases death has resulted. Tardy eruption of the temporary teeth is commonly found in rickets.

### Syphilis and the Teeth.

In hereditary syphilis premature eruption is sometimes found, but tardy eruption is probably more common. The form of the teeth is of considerable value in the diagnosis of congenital syphilis. The proper appreciation of the malformations that have a diagnostic significance depends on the recognition that the disease is a constitutional and not a local one, and that its action is manifested during the last months of intra- and the first months of extra-uterine life. The action of syphilis before the time mentioned is usually signalised by abortion, and it is for this reason that characteristic lesions of temporary teeth are so rarely seen. That they do occur, however, must not be forgotten. The tooth in the temporary dentition most frequently affected is the second molar. The teeth whose crowns calcify during the last months of intra-uterine life and the first months of extra-uterine life are the first permanent molars, the permanent incisors, and canines. These, therefore, are the teeth which should be inspected where syphilis is suspected. If malformations are found they must be symmetrical and multiple or they have no diagnostic significance. The teeth most characteristic of congenital syphilis are known as Hutchinson's teeth. The upper central incisors are most commonly affected, but a similar deformity may sometimes be observed in the lower incisors. In its typical form the tooth is broader at the neck than at the cutting edge. The cutting edge exhibits a crescentic notch formed by the rapid destruction of the thin friable tissue which bridged the deficiency at the time of eruption. Other malformations are cuspal atrophy affecting the first permanent molar and the canines, and erosions which may be sulciform or pitted. The premolars and second and third molars, calcifying as they do at a later date, are nearly always exempt.

The mouth should receive proper attention as a preliminary to operations on the stomach, and, indeed, all operations, for the removal of obvious septic foci is only a reasonable procedure. In cases of infective arthritis the condition of the mouth should be carefully investigated, and too much trust must not be placed in antiseptic mouth-washes, for pyorrhœa needs rigorous and systematic treatment.

Other conditions in which the teeth need consideration are dyspepsia, chlorotic anæmias and pernicious anæmia, ulcers of the tongue and consequent epithelioma, affections of the maxillary antrum, and middle ear disease.

